# Dr. Charles F. Durand

**Published:** 1913-12

**Authors:** 


### OBITUARIES

---

*Readers are requested to report promptly the death of all physicians in Western New York, or former residents of this region, or graduates of any medical school in Western New York and to notify the families of the deceased of our desire to publish adequate obituary notices.*

---

Dr. Charles F. Durand, Toronto, 1887, died in Toronto, Nov. 10, of pneumonia. Dr. Durand was for many years in private practice in Buffalo, and, subsequently, in charge of the isolation hospital under the direction of the department of health. Last year he moved to Toronto.

